# Characterization of the gut microbiome in Wuhuang pigs and their crossbred offspring

**DOI:** 10.3389/fvets.2025.1668076

**Published:** 2025-09-22

**Authors:** Zhijuan Yan, Yiting Yang, Xinghong Yu, Ziling Hao, Yong Du, Yan Wang, Yuanyuan Wu, Ye Zhao, Lili Niu, Xiaofeng Zhou, Linyuan Shen, Mailin Gan, Li Zhu

**Affiliations:** ^1^Farm Animal Germplasm Resources and Biotech Breeding Key Laboratory of Sichuan Province, Sichuan Agricultural University, Chengdu, China; ^2^State Key Laboratory of Swine and Poultry Breeding Industry, Sichuan Agricultural University, Chengdu, China; ^3^Key Laboratory of Livestock and Poultry Multi-omics, Ministry of Agriculture and Rural Affairs, College of Animal and Technology, Sichuan Agricultural University, Chengdu, China; ^4^Department of Biological Engineering, Sichuan Fisheries School, Chengdu, China

**Keywords:** Wuhuang pig, gut microbiota, 16S rDNA, crossbreeding, cecum, ileum, stress resistance, PICRUSt2

## Abstract

**Introduction:**

As an indigenous Chinese breed, Wuhuang pigs are valued for their stress resistance, tolerance to coarse feed, and high lean meat yield, while Berkshire pigs serve as ideal sires due to superior meat quality and early maturity. To explore the microbial basis of hybrid vigor in these breeds, we compared the gut microbiota of purebred Wuhuang pigs and Wuhuang–Berkshire hybrids.

**Methods:**

Microbial composition was assessed via 16S rDNA sequencing, and predictive functional profiling was performed using PICRUSt2 analysis.

**Results:**

Hybrids exhibited significantly increased microbial *α*-diversity and altered *β*-diversity. Notably, hybrid ceca were enriched with probiotic genera involved in fiber degradation and short-chain fatty acid (SCFA) production—such as Prevotella, Ruminococcus, Lachnospiraceae, and Roseburia—accompanied by a higher Firmicutes-to-Bacteroidetes ratio and strengthened microbial network connectivity. Predictive functional profiling further revealed significantly elevated activity in hybrid pigs for key metabolic pathways including tryptophan synthesis, pyridoxal salvage, and galacturonic acid metabolism (FDR < 0.05).

**Discussion:**

These results imply that hybrid animals leverage enriched probiotic consortia to augment nutrient metabolism and immune function, thereby supporting improved stress resilience and feed efficiency. This study provides potential microbial targets for the future genetic improvement of indigenous pig breeds.

## Graphical abstract

In this experiment, six purebred Wuhuang sows and six crossbred pigs (with purebred Wuhuang sows as the maternal parent and purebred Berkshire boars as the paternal parent) were selected. Samples of the contents from their ileum and cecum were collected. High-throughput sequencing on the Illumina platform was performed to generate metagenomic data, which was then used to construct differential microbial expression matrices. The results demonstrated that the abundance of probiotics in the intestines of the crossbred pigs was significantly higher than that in the purebred Wuhuang pigs.
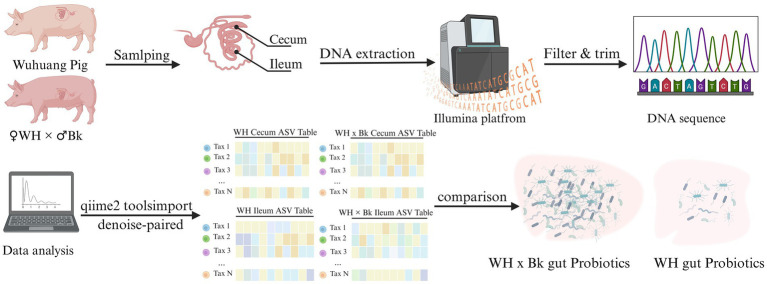

## 1 Introduction

The Wuhuang pig is a large, late-maturing indigenous breed originating from Sichuan Province, China. Identified by its elongated snout, this breed exhibits strong adaptive traits typical of Chinese local pigs, including overall robustness—encompassing stress and disease resistance—along with tolerance to coarse-feed diets, high lean meat yield, and superior reproductive performance. Historically, Wuhuang pigs faced severe population declines due to the proliferation of commercial breeds, nearly leading to extinction. Notably, it was only officially rediscovered and confirmed as a distinct genetic resource during China’s Third National Survey of Livestock and Poultry Genetic Resources, marking a critical milestone in its conservation—a process documented as the “Wuhuang Pig Rebirth” (1).

Recognizing its unique advantages, the Wuhuang pig has been the focus of ongoing efforts in systematic evaluation, conservation, and breeding utilization. Previous research has established its value through combining ability tests and crossbreeding programs, leading to breeding systems that leverage its maternal strengths—such as prolificacy, robustness, and high lean yield—within structured hybridization schemes.

While the role of the gut microbiome in shaping host phenotypes is well-established in swine production, extensive research has predominantly focused on Western commercial breeds (2). Studies on indigenous Chinese breeds are accumulating, yet they remain disproportionately centered on a few renowned breeds such as Meishan and Tibetan pigs (3). Within this context, a comprehensive, deep-sequencing analysis of the Wuhuang pig’s gut microbiome—a crucial mediator of its noted physiological resilience and metabolic efficiency—is still lacking, representing a significant knowledge gap given its distinct genetic background and valuable trait portfolio. Through the fermentation of indigestible polysaccharides, the gut microbiota produces short-chain fatty acids (SCFAs) that serve as crucial energy sources and signaling molecules, regulating key metabolic processes including energy homeostasis, lipid metabolism, and appetite (4–7). Additionally, microbial metabolites such as SCFAs and neurotransmitters mediate neuro-immune crosstalk along the gut-brain axis through direct interactions with immune cell (8–10). Concurrently, commensal microbes like *Faecalibacterium* and *Roseburia* species reinforce intestinal barrier integrity and maintain immune homeostasis via anti-inflammatory mechanisms, notably IL-10 induction and suppression of the NF-κB pathway (11, 12). Critically, these functional roles exhibit spatial compartmentalization along the gastrointestinal tract: The ileum—functioning as a site for terminal nutrient absorption and immune surveillance—is predominantly colonized by facultative anaerobes such as *Lactobacillus* and *Streptococcus* species, which metabolize residual substrates while strengthening mucosal defenses (13, 14). In contrast, the cecum operates as a specialized fermentation bioreactor dominated by strict anaerobes including Bacteroides, Prevotella, and Clostridia clusters, which drive fiber digestion (15).

To address the knowledge gap surrounding this indigenous breed, we present one of the first in-depth characterizations of the Wuhuang pig gut microbiota, with a focus on region-specific (ileum vs. cecum) community structure and functional potential using 16S rRNA gene profiling. This study not only provides foundational data on the microbial ecology of a conserved Chinese genetic resource but also advances strategies for exploiting its unique advantages in sustainable swine production.

## 2 Materials and methods

### 2.1 Sample collection and animal characteristics

In this study, we used six purebred Wuhuang castrated male pigs (barrows) and six crossbred castrated male pigs (with Wuhuang sows as the maternal line and Berkshire boars as the paternal line). All animals were raised in a commercial farm under the same controlled environmental conditions, including temperature, humidity, and feeding regimen, to ensure consistent growth conditions. All animals were raised in the same controlled-environment building on a commercial farm to ensure consistent growth conditions. The temperature was maintained at 22 ± 2 °C, and relative humidity was controlled at 65 ± 5%. Pigs were fed a standard corn-soybean meal-based diet (formulated to contain 16% crude protein and 3,100 kcal/kg digestible energy), with free access to water. All environmental and nutritional conditions were applied uniformly across both groups throughout the study period. All experimental procedures involving animals were approved by the Institutional Animal Welfare and Ethics Committee of Sichuan Agricultural University (Approval No. 20240511) and were conducted in strict compliance with national guidelines and the ARRIVE guidelines 2.0. The slaughter procedure strictly adhered to humane slaughter standards and routine operational protocols to ensure animal welfare, sample integrity, and procedural standardization. The process was performed by certified personnel in a professional slaughterhouse to minimize distress. Six purebred Wuhuang pigs and six Wuhuang-Berkshire crossbred pigs (*n* = 6 per group) were slaughtered. All animals originated from a registered breeding farm and were confirmed as sexually mature, having reached standard slaughter weight. Prior to slaughter, pigs had free access to water and feed but were fasted for 24 h with water available ad libitum. Ileal and cecal content samples were collected within 10 min post-slaughter, immediately snap-frozen in liquid nitrogen, and stored at −80 °C.

### 2.2 16S rDNA sequencing of intestinal contents

Collected intestinal content samples were sent to Novogene Co., Ltd. (Beijing, China) for total genomic DNA extraction, DNA quality assessment, library preparation, and sequencing. The V3-V4 hypervariable region of the bacterial 16S rRNA gene was amplified with primers 515F (5′-GTGYCAGCMGCCGCGGTAA-3′) and 806R (5′-GGACTACNVGGGTWTCTAAT-3′) and sequenced for each sample. Paired-end sequencing (2 × 250 bp) was performed on the Illumina NovaSeq 6,000 platform.

### 2.3 Bioinformatic analysis of 16S rDNA sequencing data

16S rDNA gene sequencing data were analyzed using QIIME 2 (version 2022.8.3). Raw paired-end reads were imported via qiime tools import. Denoising, error correction, and chimera removal were performed with qiime dada2 denoise-paired --p-trunc-len-f 230 --p-trunc-len-r 220, generating amplicon sequence variants (ASVs). Low-abundance features (frequency < 10) were filtered using qiime feature-table filter-features. A phylogenetic tree was constructed via qiime phylogeny align-to-tree-mafft-fasttree. Alpha diversity (Chao1, Shannon) and beta diversity (weighted UniFrac, Bray-Curtis) metrics were calculated using qiime diversity core-metrics-phylogenetic --p-sampling-depth 4,000. Taxonomic assignment to the genus level was performed with qiime feature-classifier classify-sklearn against the SILVA 138 reference database. ASVs were converted to Greengenes 13_5 IDs using PICRUSt2_pipeline.py. Functional potential prediction was subsequently conducted using PICRUSt2 (version 2.5.2) with default parameters.

### 2.4 Downstream statistical and visual analyses

Following functional prediction, downstream statistical analyses and visualizations were performed in R (version 4.3.1). Microbial taxonomic biomarkers were identified using LEfSe (Linear Discriminant Analysis Effect Size) analysis implemented via the microbiomeMarker package (version 1.8.0). Features with an LDA score > 3.0 and a significance threshold of *p* < 0.05 (Kruskal-Wallis test) were considered discriminative. The LEfSe results were visualized as bar plots depicting LDA scores and cladograms illustrating phylogenetic distributions of significant features across groups. Phylum-level taxonomic composition was analyzed by aggregating SILVA-classified ASVs, with the top 20 most abundant phyla visualized as stacked bar plots using ggplot2 (version 3.4.2). For genus-level analysis, a hierarchically clustered heatmap (Euclidean distance, Ward.D2 linkage) of relative abundances was generated using the pheatmap package (version 1.0.12). Co-occurrence networks for genera shared among groups ZC (purebred ileum), CC (purebred cecum), ZI (hybrid ileum), and CI (hybrid cecum) were constructed with SpiecEasi (version 1.1.5), retaining edges with Spearman *|p|* > 0.6 and a significance cutoff of *p* < 0.001 (FDR-corrected), and visualized using igraph (version 1.5.1). KEGG pathway enrichment was assessed at Levels 1 and 2; significantly enriched pathways were visualized as a heatmap (pheatmap). Differential metabolic pathway analysis for specific comparisons—CC vs. CI, ZC vs. ZI, CC vs. CI (validation), and CC vs. ZC—was performed using DESeq2 (version 1.40.2) with a negative binomial model. Log₂ fold changes were estimated using the apeglm shrinkage estimator to improve the accuracy and reliability of effect sizes. Differential abundance was assessed using an FDR-adjusted *p* < 0.05.

## 3 Results

### 3.1 Overview of trait statistics and data quality

**Figure 1:**
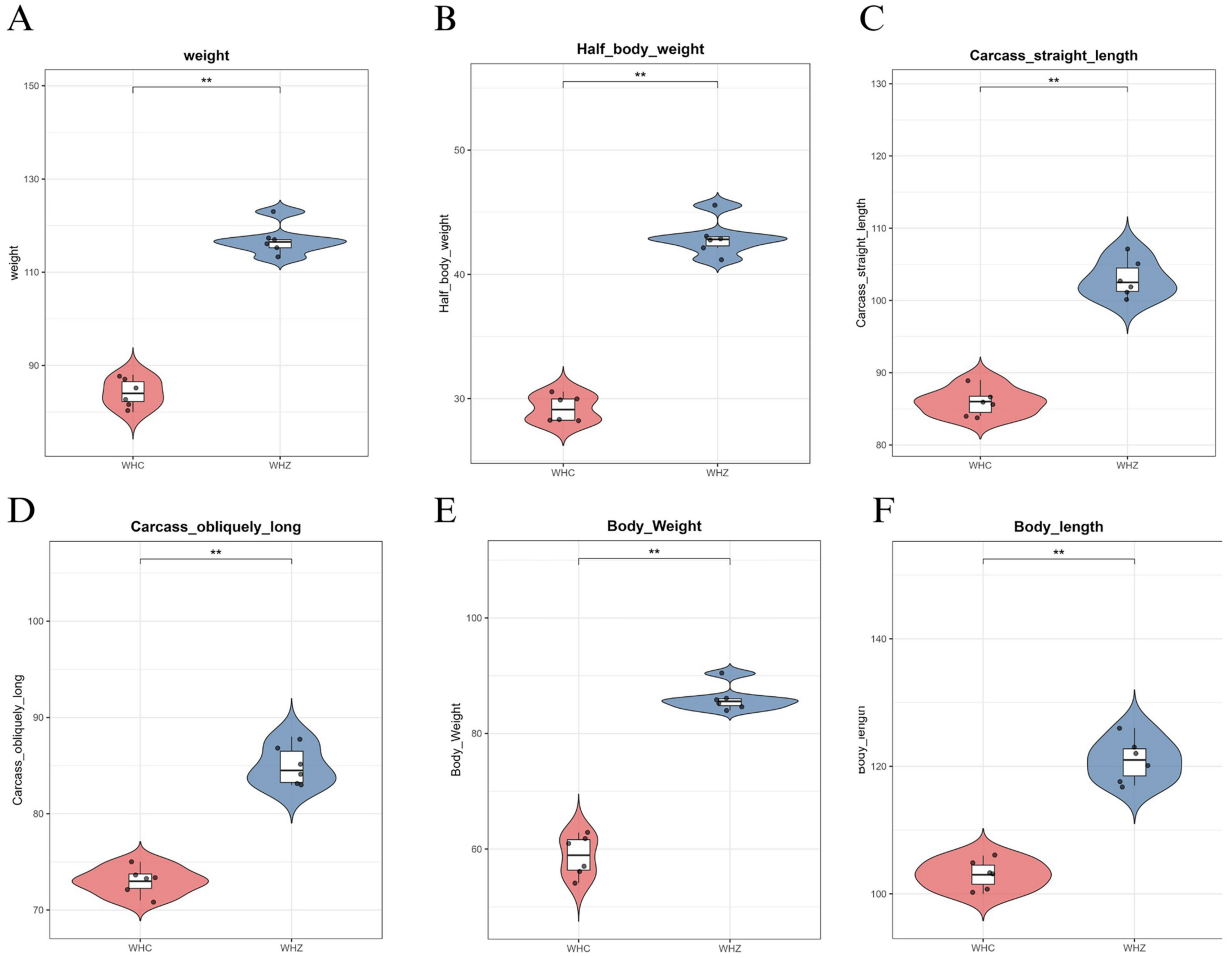
**(A)** Live weight, **(B)** Half-carcass weight, **(C)** Carcass straight length, **(D)** Carcass oblique length, **(E)** Whole-body weight, **(F)** Body length. Data represent mean ± SEM (*n* = 6). Statistical significance denoted by *p < 0.05, **p < 0.01, ***p < 0.001 (two-tailed *t*-test). WHZ hybrids demonstrate significantly enhanced growth metrics and carcass yields compared to WHC purebreds, particularly in weight-related parameters **(A,B,E)** and dimensional traits **(C,D,F)**, indicating heterosis effects on economically important production characteristics.

**Figure 2:**
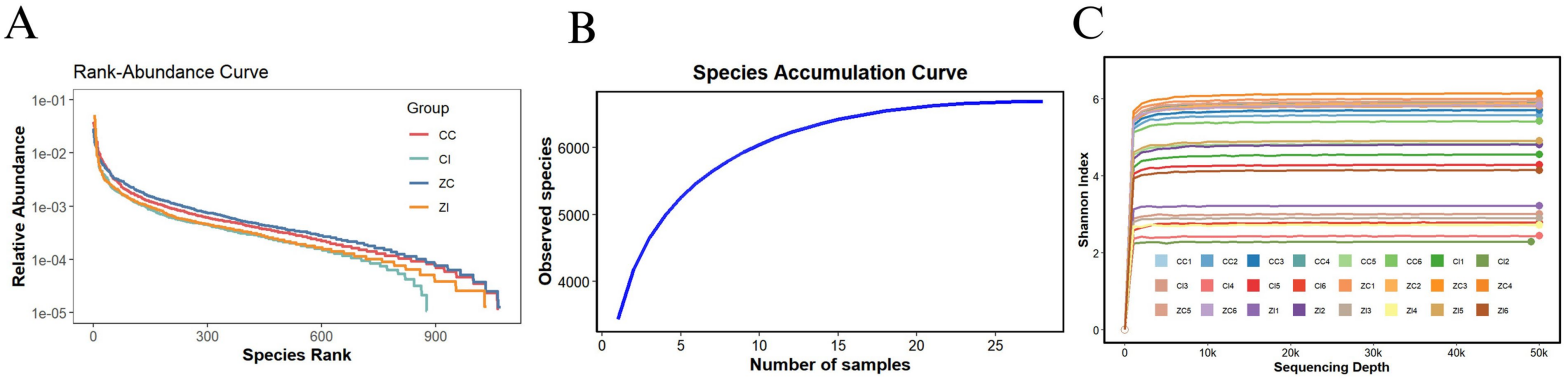
**(A)** Rank-abundance curve: The x-axis represents the species rank (ordered by decreasing relative abundance), and the y-axis shows the relative abundance on a logarithmic scale. **(B)** Species accumulation curve: This panel displays the species accumulation curve, indicating the number of observed species as a function of the number of samples. The x-axis denotes the number of samples, while the y-axis represents the count of observed species. **(C)** Shannon diversity index curve: This panel presents the Shannon diversity index plotted against sequencing depth. The x-axis shows the sequencing depth, and the y-axis indicates the Shannon index value.

According to measurements of body weight and dimensions, adult Wuhuang pigs demonstrate significantly lower body weight and smaller physique compared to Wuhuang-Berkshire Hybrid Pigs, highlighting a considerable contrast between these two breeds (Figures 1A–F). To investigate whether the phenotypic differences are associated with variations in the intestinal microbiota characteristics of the ileum and cecum, 16S rDNA sequencing was performed on the ileal and cecal contents of the two pig breeds. The rank-abundance curve (Figure 2A) delineates species distribution hierarchies across experimental groups (CC, CI, ZC, ZI), revealing community structure through logarithmic relative abundance patterns (10^−1^–10^−5^) where curve architecture identifies dominant taxa while the extended tail signifies rare biosphere contributions, with annotated diversity indices quantifying heterogeneity. Complementing this, the species accumulation curve (Figure 2B) demonstrates asymptotic saturation of observed species richness with increasing sampling effort, where plateau formation validates sampling adequacy by indicating diminishing returns in new species discovery. Concurrently, the Shannon diversity progression (Figure 2C) tracks *α*-diversity stabilization across sequencing depths (0-500 k reads), with sample-specific trajectories transitioning from nonlinear increases to plateaus that confirm sufficient sequencing depth for reliable diversity quantification, as the Shannon index integrates both species richness and evenness. Collectively, these orthogonal analyses establish methodological rigor: the rank-abundance curve characterizes fundamental community organization, the accumulation curve verifies comprehensive species capture, and the depth-dependent diversity profiles ensure robust measurement validity, thereby providing integrated quality assessment for subsequent comparative ecological analyses of microbial community dynamics.

### 3.2 Significant differences in cecal microbiota diversity between purebred Wuhuang and Wuhuang-Berkshire hybrid pigs

**Figure 3:**
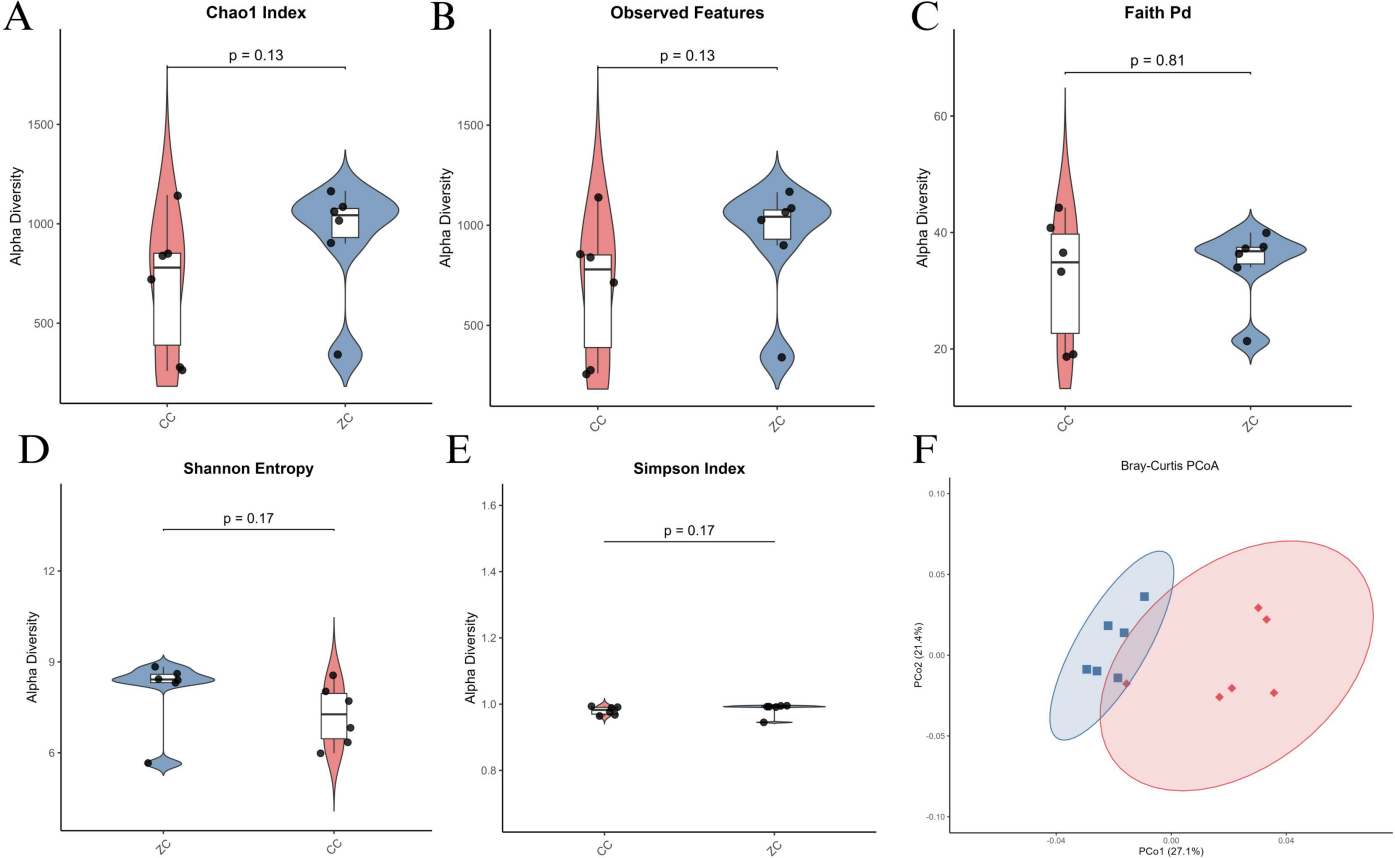
Alpha and beta diversity analysis of cecal microbiota in purebred Wuhuang vs. Wuhuang-Berkshire hybrid pigs. **(A-E)** Alpha diversity indices: Chao1 Index, Observed Features, Faith’s PD, Shannon Entropy, and Simpson Index. **(F)** Principal Coordinates Analysis (PCoA) of beta diversity based on Bray–Curtis dissimilarity (*T*-test, *p* < 0.001). Ellipses represent 99% confidence intervals.

Alpha diversity analysis revealed no significant differences in the Chao1 Index (Figure 3A) and Observed Features (Figure 3B) between the two groups, with *p*-values of 0.13 and 0.13 respectively, suggesting similar species richness. However, Faith’s PD (Figure 3C) indicated a trend toward higher phylogenetic diversity in the Purebred group (*p* = 0.81), though not statistically significant. Further, Shannon Entropy (Figure 3D) and Simpson Index (Figure 3E) showed greater diversity in the Purebred group (*p* = 0.17 and *p* = 0.17 respectively), highlighting differences in community evenness. Beta diversity analysis via Bray-Curtis PCoA (Figure 3F) demonstrated distinct clustering patterns, with significant separation between the groups, underscoring differences in microbial community structure. Collectively, these results indicate that while species richness was comparable, the Purebred Wuhuang group exhibited greater diversity and evenness, alongside distinct microbial community composition compared to the Hybrid group, particularly within the cecal microbiota.

### 3.3 Significant differences in ileal microbiota diversity between purebred Wuhuang and Wuhuang-Berkshire hybrid pigs

**Figure 4:**
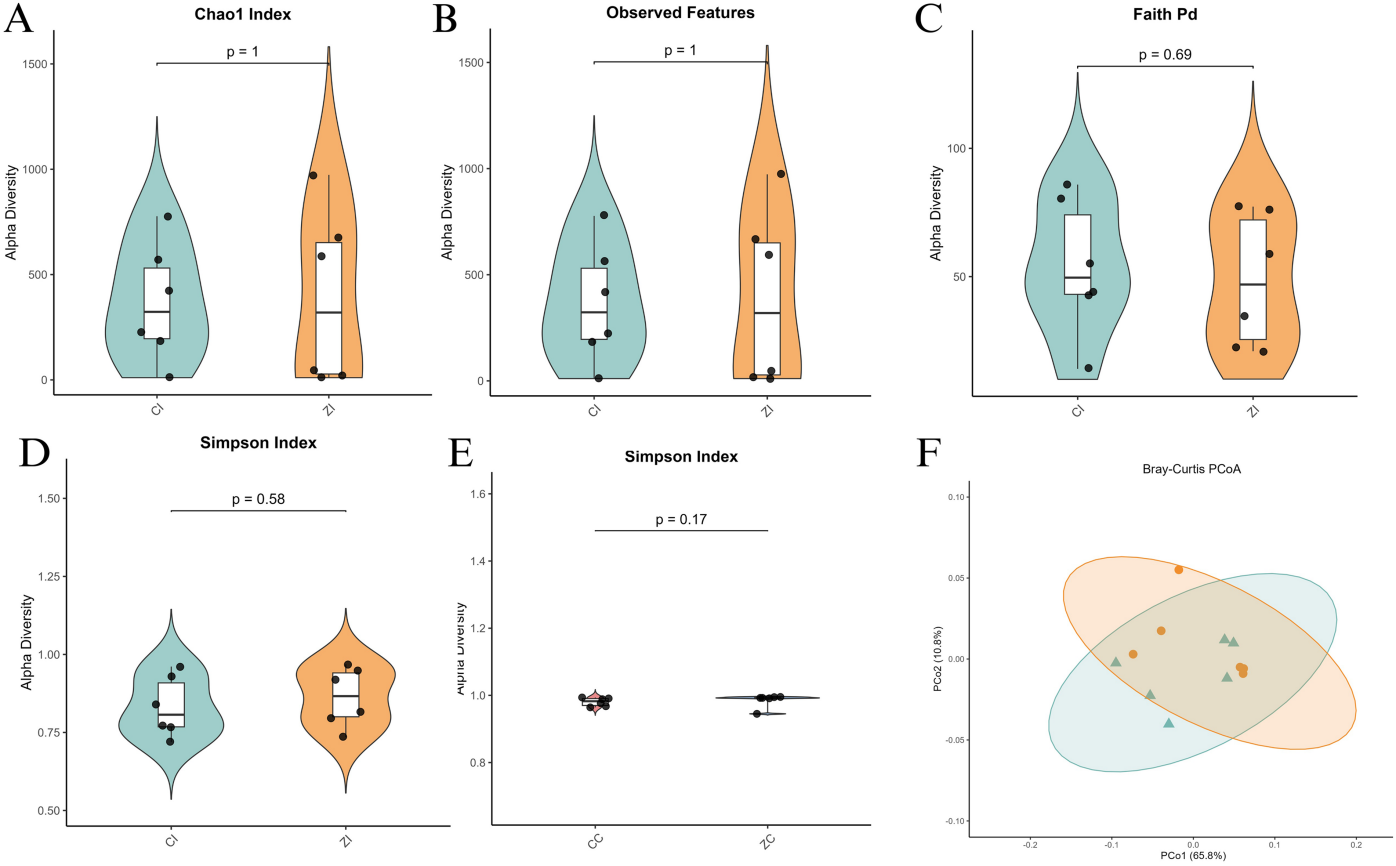
Alpha and beta diversity analysis of ileal microbiota in purebred Wuhuang vs. Wuhuang-Berkshire hybrid pigs. **(A-E)** Alpha diversity indices: Chao1 Index, Observed Features, Faith’s PD, Shannon Entropy, and Simpson Index. **(F)** Principal Coordinates Analysis (PCoA) of beta diversity based on Bray–Curtis dissimilarity (*T*-test, *p* < 0.001). Ellipses represent 99% confidence intervals.

Similar analyses were conducted on the ileal microbiota. Alpha diversity metrics, including the Chao1 Index, Observed Features, Faith’s PD, Shannon Entropy, and Simpson Index, revealed comparable species richness between the two groups, with no significant differences in Chao1 Index (*p* = 1) and Observed Features (*p* = 1) (Figures 4A–C). However, the Shannon Entropy (*p* = 0.58) and Simpson Index (*p* = 0.17) indicated greater diversity and evenness in the Purebred Wuhuang group (Figures 4D,E). Beta diversity analysis through Bray-Curtis PCoA also demonstrated distinct clustering and significant separation between the groups, reflecting differences in microbial community structure (Figure 4F). Collectively, these findings suggest that the Purebred Wuhuang pigs exhibit a more diverse and even ileal microbiota compared to the Hybrid group, consistent with the cecal microbiota analysis.

### 3.4 Integrated analysis of cecal microbiota composition and functional biomarkers

**Figure 5:**
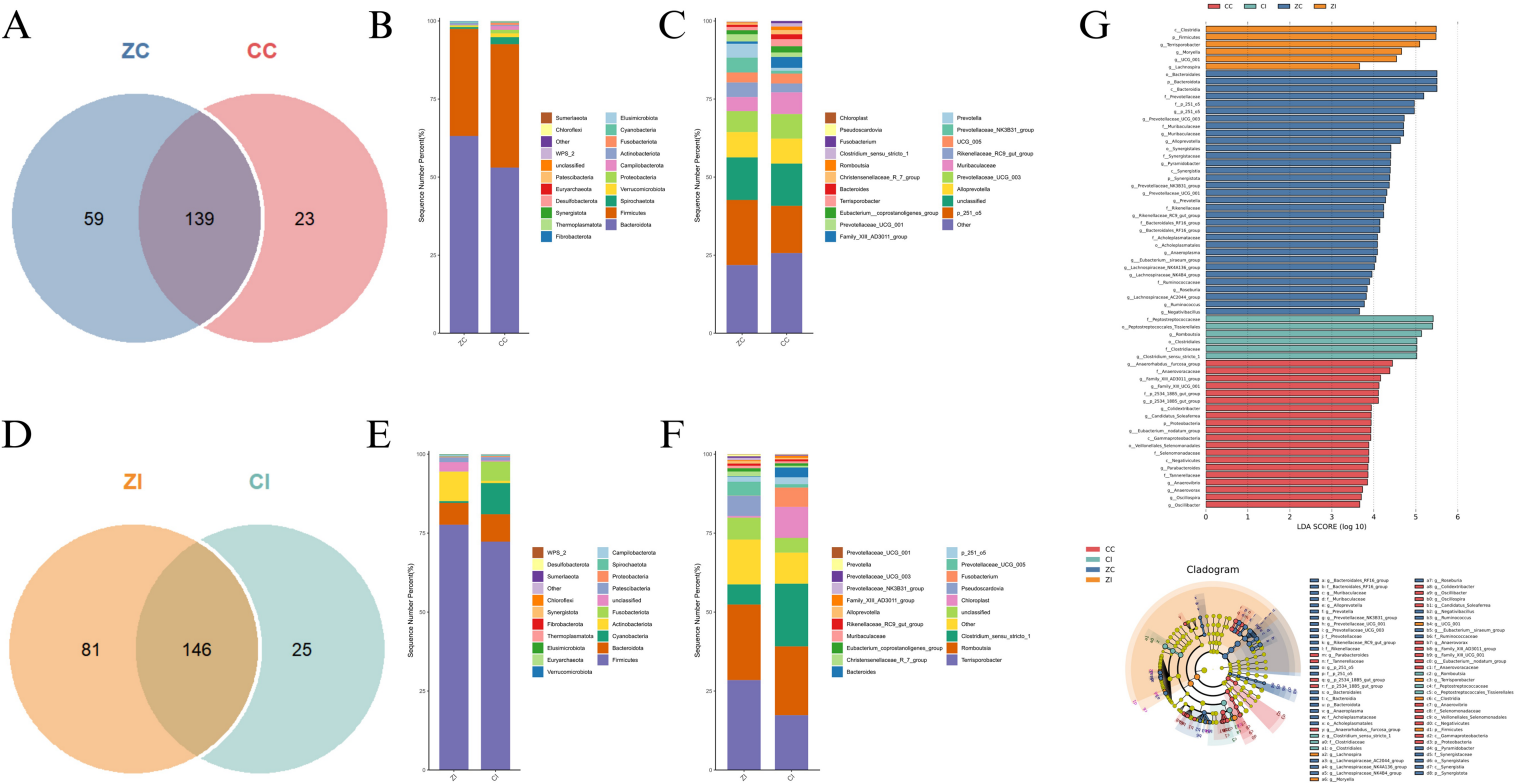
**(A)** Core microbiome Venn diagram comparing purebred (CC) and hybrid (CI) cecal communities. **(B)** Phylum-level taxonomic distribution showing dominant bacterial lineages. **(C)** Genus-level composition of the top 20 taxa, highlighting differential abundance patterns. **(D)** Shared and unique OTUs between purebred (ZC) and hybrid (ZI) ileal microbiomes. **(E)** Phylum-level community structure in ileal niches. **(F)** Genus-resolution taxonomic profile of predominant ileal microorganisms. **(G)** LEfSe biomarker identification: Significantly enriched taxa between intestinal sites (LDA score > 3.0, *p* < 0.05) visualized through bar plot (left) and cladogram (right), with Lakkoeff test kit validation confirming metabolic functional differences. Group designations: CC, purebred cecum; CI, hybrid cecum; ZC, purebred ileum; ZI, hybrid ileum.

The cecal microbiota exhibited significant compositional divergence between purebred Wuhuang and Wuhuang-Berkshire hybrid pigs, with core microbiome analysis revealing 139 shared OTUs alongside 23 and 59 unique operational taxonomic units in CC and ZC, respectively, (Figure 5A), indicating substantial breed-specific microbial signatures. Taxonomic profiling demonstrated *Firmicutes* and *Bacteroidetes* dominance at the phylum level (Figure 5B), though ZC displayed a 1.8-fold higher Firmicutes-to-Bacteroidetes ratio driven primarily by enriched *Lactobacillaceae* populations. At genus resolution (Figure 5C), ZC harbored significantly elevated proportions of butyrate-producing *Megasphaera* and starch-metabolizing *Prevotella_9*, while CC showed exclusive enrichment of cellulose-degrading *Ruminococcaceae_UCG-005*. The genus-level analysis also revealed that ZC had significantly higher relative abundance of *Megasphaera* and *Prevotella_9*, which are associated with butyrate production and starch metabolism respectively, while CC showed exclusive enrichment of *Ruminococcaceae_UCG-005*, which is linked to cellulose degradation. LEfSe biomarker analysis (Figure 5G) identified distinct microbial taxa as biomarkers across different groups. The analysis showed that certain taxa were enriched in specific groups, with some displaying higher LDA scores. For instance, taxa such as *c_Clostridia*, *p_Firmicutes*, and *g_Terrisporobacter* were enriched in the CC group, while *o_Bacteroidales*, *p_Bacteroidota*, and *f_Prevotellaceae* were enriched in the ZC group. These results highlight the differences in microbial composition between the groups.

### 3.5 Comparative analysis of ileal microbiota composition and biomarkers

Ileal microbial communities demonstrated pronounced structural and functional distinctions between purebred and hybrid pigs, beginning with core microbiome analysis that identified 146 conserved OTUs while revealing 25 and 81 unique operational taxonomic units in CI and ZI, respectively, (Figure 5D), indicating greater microbial niche specialization in the hybrid ileum. Taxonomic stratification revealed Proteobacteria dominance in both groups (Figure 5E), but ZI exhibited a 2.3-fold enrichment in *Actinobacteria* concomitant with reduced *Bacteroidetes* prevalence. At genus resolution (Figure 5F), ZI harbored significantly elevated *Lactobacillus* populations and *Streptococcus* proportions, whereas CI maintained higher *Clostridium_sensu_stricto_1* abundance. LEfSe biomarker mapping (Figure 5G) confirmed *Lactobacillus* and *Bifidobacterium* as signature ZI taxa (LDA > 4.2).

Comparative analysis at the genus level revealed significant enrichment of putative probiotic taxa—including *Muribaculaceae, Pyramidobacter, Prevotella,* Rikenellaceae_RC9_gut_group, *Roseburia*, *Ruminococcus, Bacteroidales_RF16_group, Eubacterium_siraeum_group,* Lachnospiraceae_NK4B4_group and among others (16–20)—in the cecum of hybrid pigs compared to purebred Wuhuang pigs.

### 3.6 Functional and ecological dynamics in cecal microbiota

**Figure 6:**
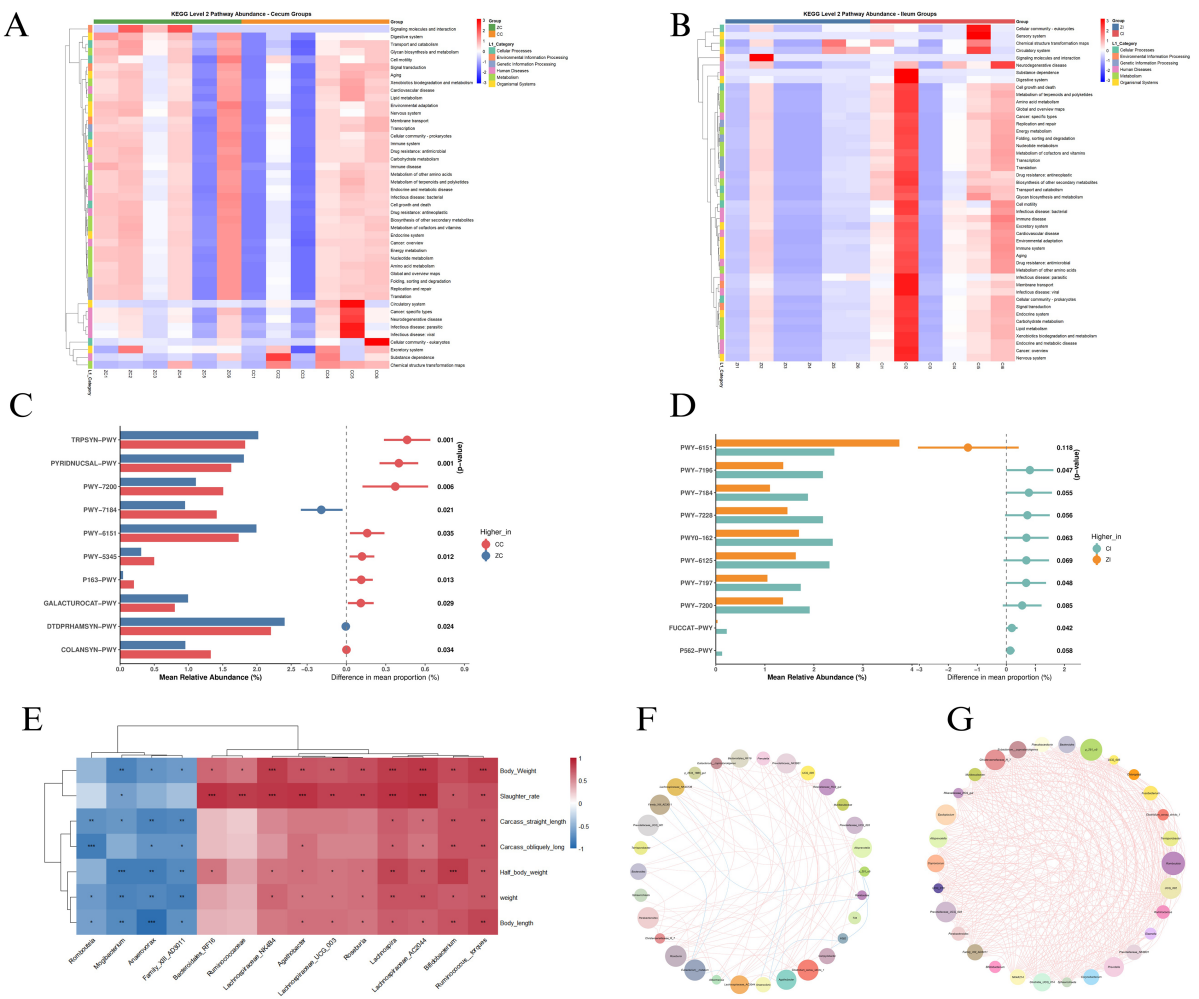
**(A)** KEGG pathway heatmap of cecal microbiota functions in purebred (CC) vs. hybrid (ZC) pigs. **(B)** KEGG pathway heatmap of ileal microbiota functions in purebred (CI) vs. hybrid (ZI) pigs. **(C)** Metabolic pathway enrichment analysis of cecal microbiota (ZC vs. CC). **(D)** Metabolic pathway enrichment analysis of ileal microbiota (ZI vs. CI). **(E)** Spearman correlation matrix between microbial genera and growth/carcass traits: weight, body length, carcass straight length, carcass oblique length, body weight, half body weight, slaughter rate. **(F)** Cecal microbial co-occurrence network topology. **(G)** Ileal microbial co-occurrence network architecture.

The cecal functional landscape revealed fundamental metabolic distinctions between genotypes. KEGG pathway analysis at level 2 (Figure 6A) demonstrated significant enrichment of carbohydrate metabolism pathways in hybrid pigs (ZC). Metabolic reconstruction (Figure 6C) identified distinct pathway abundance profiles, with hybrid pigs (ZC) exhibiting enhanced representation of pyridine nucleotide salvage pathways (PYRIDNUCSAL-PWY) and galacturonate catabolism (GALACTUROCAT-PWY), while purebreds (CC) showed predominant expression of tryptophan biosynthesis (TRPSYN-PWY) and colanic acid building blocks biosynthesis (COLANSYN-PWY). Ecological network analysis (Figure 6F) revealed distinct topological organizations in the cecal microbiota, with hybrid pigs (ZC) exhibiting a modular architecture centered on *Prevotella* as a primary hub, demonstrating strong co-occurrence linkages with *Prevotellaceae_UCG_001* and *Lachnospiraceae_NK4A126*. This core consortium showed complementary functional associations with *Roseburia* and *Anaerovibrio*, forming a putative polysaccharide-degrading guild. Conversely, purebreds (CC) displayed Bacteroides-centric clustering with *Parabacteroides* and *Rikenellaceae_RC9_gut*, indicative of protein-centric metabolic strategies.

### 3.7 Ileal functional adaptations and host phenotype integration

Ileal functional profiling revealed compartment-specific adaptations in metabolic priorities, where hybrid pigs (ZI) exhibited significant enrichment in carbohydrate metabolism and lipid metabolism pathways (Figure 6B), contrasting with purebred (CI) dominance of amino acid metabolism and drug resistance pathways. This metabolic divergence was particularly evident in the enhanced representation of glycan biosynthesis and energy metabolism modules in hybrids, while purebreds showed preferential expression of nucleotide metabolism and xenobiotics biodegradation pathways. Metabolic pathway analysis (Figure 6D) identified distinct ileal functional profiles, with hybrid pigs (ZI) exhibiting enhanced representation of fucose and rhamnose degradation pathways (FUCCAT-PWY and P562-PWY), alongside polyamine biosynthesis modules (PWV-7196 and PWV-7184). Conversely, purebreds (CI) showed predominant expression of aromatic compound degradation pathways (PWY-6151 and PWY-7228) and cofactor salvage systems (PWY0-162). Phenotype-microbiota integration (Figure 6E) revealed significant correlations across various microbial taxa and host traits. Notably, ileal Lactobacillus abundance showed a strong positive correlation with slaughter rate, while Bifidobacterium levels were significantly associated with body length. Additionally, several other microbial taxa exhibited significant correlations with different host phenotypic traits, indicating a complex interplay between the gut microbiota and host characteristics. Co-occurrence networks (Figure 6G) revealed distinct architectural differences, with hybrids exhibiting simplified networks characterized by *Lactobacillus-Streptococcus* mutualism and a reduced average path length. These features suggest enhanced metabolic efficiency, potentially facilitating nutrient absorption. This is supported by positive correlations between network density and weight gain metrics, indicating that the streamlined network architecture in hybrids may contribute to improved growth performance.

## 4 Discussion

The Wuhuang pig, an indigenous Chinese breed, exhibits robust stress resistance and remarkable tolerance to coarse-feed diets. Compared to purebred Wuhuang pigs, Wuhuang-Berkshire hybrids (with Berkshire sires and Wuhuang dams) demonstrate enhanced environmental adaptability and higher lean meat yield (21). Our comparative analysis of ileal and cecal microbiota reveals significant hybridization effects on gut microbial architecture and metabolic potential, with hybrids exhibiting elevated *α*-diversity and restructured *β*-diversity in both intestinal segments—particularly pronounced in the cecum. These findings align with the well-documented heterosis phenomenon in swine crossbreeding (22), wherein enhanced host genetic diversity (e.g., MHC polymorphism) remodels gut ecosystems through immune-microbe crosstalk (23). The enrichment of fiber-degrading genera like *Prevotella* and *Roseburia* in hybrid ceca (24) provides a microbiological basis for improved fiber utilization, while proliferation of probiotics including Muribaculaceae and Lachnospiraceae underpins intestinal homeostasis through butyrate-mediated barrier fortification (25) and Prevotella-driven Th17/Treg balance regulation (26). Notably, the altered *Firmicutes/Bacteroidetes* ratio in hybrid cecum resonates with Backhed’s “energy harvest” theory (27), potentially explaining superior feed conversion efficiency via increased SCFA production. Conversely, abnormal Cyanobacteria enrichment in purebred ileum—reported in stressed swine (28)—may indicate heightened environmental sensitivity.

We confirm distinct metabolic compartmentalization along the gut: the ileum (primary nutrient absorption site) shows enhanced tryptophan biosynthesis (TRPSYN-PWY) and galacturonate metabolism (GALACTUROCAT-PWY) in hybrids, aligning with its facultative anaerobe-dominated microbiota. Tryptophan metabolites like kynurenine activate aryl hydrocarbon receptor (AhR) pathways to regulate gut immunity (29), potentially boosting disease resistance (30). Meanwhile, the cecum (fiber fermentation hub) exhibits upregulated collagen biosynthesis (COLANSYN-PWY) in purebreds, indicating heightened mucosal repair demands consistent with lower *α*-diversity—a functional specialization echoing Deschasaux’s “gut microbial biogeography” concept (31). Co-occurrence networks further revealed Prevotella and Lachnospiraceae as highly connected hubs in hybrids versus peripherally positioned Epulopiscium, suggesting enhanced ecological stability (32) that may confer resilience to environmental perturbations. The Berkshire genetic contribution likely influences microbiota through early-maturity traits modulating insulin-like growth factors (33) and intramuscular fat-associated genes (e.g., *FABP4*) altering bile acid profiles (34).

Functional profiling via KEGG pathway enrichment revealed a predominance of core metabolic processes within the porcine gut microbiome, most notably Carbohydrate Metabolism, Amino Acid Metabolism, and Energy Metabolism, reflecting the microbial community’s fundamental role in energy harvesting and nutrient assimilation. Concurrently, significant enrichment was observed for pathways governing microbial cellular maintenance and environmental adaptation, including Genetic Information Processing (Replication and repair, Transcription, Translation), Cellular Processes (Transport and catabolism, Folding/sorting/degradation), and Biosynthesis of Glycans, Vitamins, Cofactors, Terpenoids and Polyketides. The prominence of Membrane Transport and Signal Transduction pathways underscores sophisticated mechanisms for environmental sensing and substrate acquisition essential for microbial survival in the dynamic intestinal niche. Critically, pathways implicated in host–microbe interactions were robustly represented, encompassing Signaling Molecules and Interaction, Immune System functions, and multiple Infectious Disease modules (bacterial, viral, parasitic), indicating active microbiota-host dialogue influencing immunity and barrier homeostasis. The co-enrichment of Xenobiotics Biodegradation and Metabolism with Drug Resistance pathways (antimicrobial and antineoplastic) further suggests adaptive detoxification capabilities, potentially responsive to dietary or xenobiotic challenges. The detection of Global and Overview Maps—which represent integrated metabolic networks—confirms system-level coordination of these processes, collectively depicting a functionally synergistic microbiome optimized for nutrient metabolism, environmental resilience, and host crosstalk within the porcine gastrointestinal ecosystem.

## 5 Conclusion

This study revealed that hybrid pigs exhibit an altered gut microbiota structure with probiotic enrichment and enhanced metabolic functions—including amino acid, vitamin, and carbohydrate metabolism—which collectively contribute to improved nutrient utilization, immune modulation, and stress resistance. These findings elucidate key microbial mechanisms underlying hybrid advantages in swine. However, the inferred metabolic functions require further validation via metagenomic or metabolomic approaches. Future work will focus on causal validation of these microbial functions and their application in optimizing feed efficiency and health in pig breeding.

## Data Availability

The original contributions presented in the study are publicly available. This data can be found here: NCBI Sequence Read Archive (SRA), BioProject Accession Number: PRJNA1328342 https://www.ncbi.nlm.nih.gov/sra/PRJNA1328342.
